# The effect of yogic breathing (Pranayama) on heart rate and blood pressure in patients with hypertension: A systematic review and meta-analysis

**DOI:** 10.1016/j.ihj.2026.01.004

**Published:** 2026-01-22

**Authors:** Yogapriya Chidambaram, Pavitraa Saravana Kumar, Swetharajan Gunasekar, Laxminarasimha Reddy Kundoor, Maheshkumar Kuppusamy, Madhesh Kasi, Ambalam M. Chandrasekaran, Sadhanandham Shanmugasundaram, Sankaran Ramesh, Thanikachalam Sadagopan, Nitish Naik, Ambuj Roy, Dorairaj Prabhakaran, Nagendra Boopathy Senguttuvan

**Affiliations:** a Department of Clinical Research, Sri Ramachandra Institute of Higher Education and Research (SRIHER), No.1, Ramachandra Nagar, Porur, Chennai, Tamil Nadu, 600116, India; b Department of Human Genetics, Sri Ramachandra Institute of Higher Education and Research (SRIHER), No.1, Ramachandra Nagar, Porur, Chennai, Tamil Nadu, 600116, India; c Department of Cardiology, Sri Ramachandra Institute of Higher Education and Research (SRIHER), No.1, Ramachandra Nagar, Porur, Chennai, Tamil Nadu, 600116, India; d Department of Physiology and Biochemistry, Government Yoga and Naturopathy Medical College and Hospital, Chennai, Tamil Nadu, India; e Centre for Chronic Disease Control (CCDC), New Delhi, Delhi, 110016, India; f Department of Cardiology, All India Institute of Medical Sciences, New Delhi, Delhi, 110029, India

**Keywords:** Blood pressure, Heart rate, Heart rate variability, Prehypertension, Hypertension, Non-pharmacological intervention, Pranayama

## Abstract

**Introduction:**

Systemic hypertension is a major contributor to global cardiovascular morbidity and mortality. Effective management combines pharmacological and lifestyle interventions. Pranayama, a yogic breathing technique, may modulate cardiovascular function, but its impact on hypertension remains uncertain.

**Methods:**

A systematic review and meta-analysis following PRISMA guidelines (PROSPERO: CRD42024597502) included RCTs from PubMed, Cochrane Library, Scopus, and ClinicalTrials.gov up to April 2025. We assessed Risk of bias using Cochrane RoB 2, and pooled effects were reported as standardized mean differences (SMDs) with 95 % confidence intervals (CIs).

**Results:**

Seven RCTs (683 participants) showed a significant reduction in the primary outcome, heart rate (SMD = −0.43; 95 % CI: −0.52 to −0.34; I^2^ = 0 %). Systolic BP decreased significantly under fixed-effects (SMD = −0.72), while diastolic BP and HRV improvements were modest.

**Conclusion:**

Pranayama lowers heart rate and may modestly reduce blood pressure, supporting its role as a safe, adjunctive therapy pending larger standardized trials.

## 1 Introduction

Systemic hypertension (HTN) affects an estimated 1.28 billion adults aged 30–79 years worldwide.^1^ Of these ∼1.3 billion individuals, approximately 82 % reside in low- and middle-income countries (LMICs), with India alone accounting for over 220 million adults living with HTN.^2^ According to the Great India Blood Pressure Survey (GIBPS), approximately one in three adults (30.7 %) in India had hypertension.^3^ Although contemporary guidelines emphasize lifestyle modifications and antihypertensive medications as the first-line therapy for blood pressure management, global health data indicate gaps and disparities in blood pressure treatment and control in LMICs^4^, highlighting the need for adopting adjunctive interventions alongside conventional therapies for achieving sustained blood pressure regulation. The growing interest in safe and accessible adjunctive therapies is bolstered by the fact that patients taking antihypertensives, often for prolonged periods, may encounter issues with medication adherence due to side effects, purchasing costs, and polypharmacy. Therefore, low-cost, culturally acceptable, and feasible non-pharmacological interventions could be considered as a viable alternative. Recent comparative evidence of non-pharmacological interventions indicates that, in addition to dietary modifications, several mind-body interventions and lifestyle changes could achieve clinically meaningful reductions in blood pressure. In one large Bayesian network meta-analysis of 126 randomized controlled trials (RCTs) evaluating 22 non-pharmacological interventions, researchers found that yoga, meditation, and breathing control techniques exhibited superior efficacy compared to usual care in reducing both systolic (SBP) and diastolic blood pressure (DBP) among individuals with prehypertension and established hypertension.^5^ Other analyses and systematic reviews have reinforced the blood pressure-lowering potential of yoga-based programs, but these syntheses typically aggregated heterogeneous interventions that combined postures, relaxation, and breathing practices^6^, ^7^, ^8^. Indeed, in an updated systematic review and meta-analysis of 30 RCTs, it was found that participants assigned to yoga-based interventions achieved SBP and DBP reductions of 4.16 mmHg and 1.8 mmHg, respectively, compared to standard-of-care^7^. However, the authors noted that the included studies did not delineate the individual contributions of breathing vs posture control vs specific asanas in reducing blood pressure. Since it remains unclear which specific component, or synergistic combination, is primarily responsible for any observed cardiovascular benefits, we sought to isolate the efficacy of a discrete yogic breathing technique known as pranayama. Unlike conventional breathing exercises, pranayama encompasses a mix of techniques such as slow, deep diaphragmatic breathing, alternate nostril breathing, and rhythmic breath retention, and has been found to exert antihypertensive effects by shifting autonomic balance toward parasympathetic dominance, dampening sympathetic outflow, improving baroreflex sensitivity, and enhancing heart rate variability^9^, ^10^, ^11^. Further, the meditative and calming component inherent to pranayama may facilitate psychophysiological relaxation and stress mitigation that might also combat any elevations in blood pressure. Preliminary evidence suggests that various forms of breathing exercises, including pranayama, can modestly, yet significantly, reduce both SBP and DBP by 7.06 mmHg and 3.43 mmHg, respectively.^12^ Other studies have also demonstrated similar reductions, ranging from 2 to 10 mmHg for SBP and 1 mmHg for DBP reduction in acute settings and 4–21 mmHg for SBP and 4–7 mmHg for DBP reduction in chronic, long-term studies^13^. Despite this, evidence supporting the efficacy of pranayama remains inconclusive, and prior systematic reviews and meta-analyses have failed to quantify the independent contribution of pranayama to blood pressure reduction, since they focused on composite interventions that combined other yoga practices with breathing^7^^,^^14^ or looked at other forms of breathing along with pranayama.^12^ Therefore, we conducted a systematic review and meta-analysis restricted exclusively to RCTs examining the effects of pranayama in patients with hypertension, with the aim of clarifying its overall efficacy and role as an independent modulator of blood pressure and heart rate changes.

## 2 Methods

### 2.1 Data sources and search strategy

This systematic review and meta-analysis followed the Preferred Reporting Items for Systematic Reviews and Meta-Analyses (PRISMA) guidelines. The protocol was registered with PROSPERO and can be found online under the registration number CRD42024597502. A comprehensive literature search was conducted across PubMed, Cochrane Library, Scopus, and ClinicalTrials.gov databases up to 30th April 2025. Search terms included: "blood pressure," "hypertension," "prehypertension," "elevated blood pressure," "systolic blood pressure," "diastolic blood pressure," "mean arterial pressure," "pranayama," "bhramari pranayama," "alternate nostril breathing," and "cooling pranayama," using Boolean operators "AND" and "OR." The search was restricted to English-language studies.

### 2.2 Study selection

Studies were selected based on adherence to the following PICO (Population, Intervention, Comparator, Outcomes) framework:

• **Population:** Adults (>18 years) with elevated blood pressure (systolic blood pressure = 120–129 mmHg or diastolic blood pressure <80 mmHg), Stage 1 hypertension (systolic blood pressure = 130–139 mmHg or diastolic blood pressure = 80–89 mmHg), or Stage 2 hypertension (systolic blood pressure ≥140 mmHg or diastolic blood pressure ≥90 mmHg).
• **Intervention:** Any form of pranayama (e.g., alternate nostril breathing, cooling pranayama, bhramari pranayama). Only RCTs in which pranayama was the primary standalone intervention were included.
• **Comparator:** Standard-of -care or waitlist control groups
• **Primary Outcomes:** Heart rate
• **Secondary Outcomes:** Changes in systolic blood pressure and diastolic blood pressure. Heart rate variability such as LF - low-frequency; HF - high-frequency, NN50 - The number of pairs of successive NN (R–R) intervals that differ by more than 50 ms; pNN50 - proportion derived by dividing NN50 by the total number of NN intervals; SDNN - standard deviation of RR Intervals; RMSSD - the square root of the mean of the sum of the squares of differences between adjacent normal-to-normal (NN) intervals.

**Exclusion Criteria:** Studies utilizing interventions other than pranayama, studies not evaluating changes in SBP and DBP, observational studies, case reports, case series, qualitative studies, research protocols, unpublished theses or dissertations, abstracts, letters to the editor, and non-English publications were excluded. Trials combining pranayama with yoga postures, meditation, or lifestyle interventions were excluded, since they did not allow for isolation of pranayama-specific effects.

Two authors (YC, PSK) independently screened studies for eligibility, and discrepancies were addressed through discussion with a third co-author (NBS) and resolved by consensus.

### 2.3 Data extraction and quality assessment

Two authors (YC and PSK) independently extracted data, including basic study characteristics, participant information, intervention details, and outcome data. The Cochrane Risk of Bias 2 (RoB 2) tool was used to assess methodological quality across five domains: randomization process, deviations from intended interventions, missing outcome data, measurement of the outcome, and selection of the reported result. Wherever there was a discrepancy, a third author (NBS) resolved the issue.

### 2.4 Data synthesis and analysis

Meta-analysis was conducted using standardized mean differences (SMD) with 95 % confidence intervals (CI). Both fixed-effects (common effects) and random-effects models were employed. Heterogeneity was assessed using the I^2^ statistic, with values > 75 % indicating substantial heterogeneity. Subgroup analyses were conducted when appropriate based on intervention type and duration. Meta-regression was not performed due to the limited number of included studies, inconsistent reporting of intervention characteristics, and insufficient statistical power.

## 3 Results

### 3.1 Study selection and characteristics

**Fig. 1:**
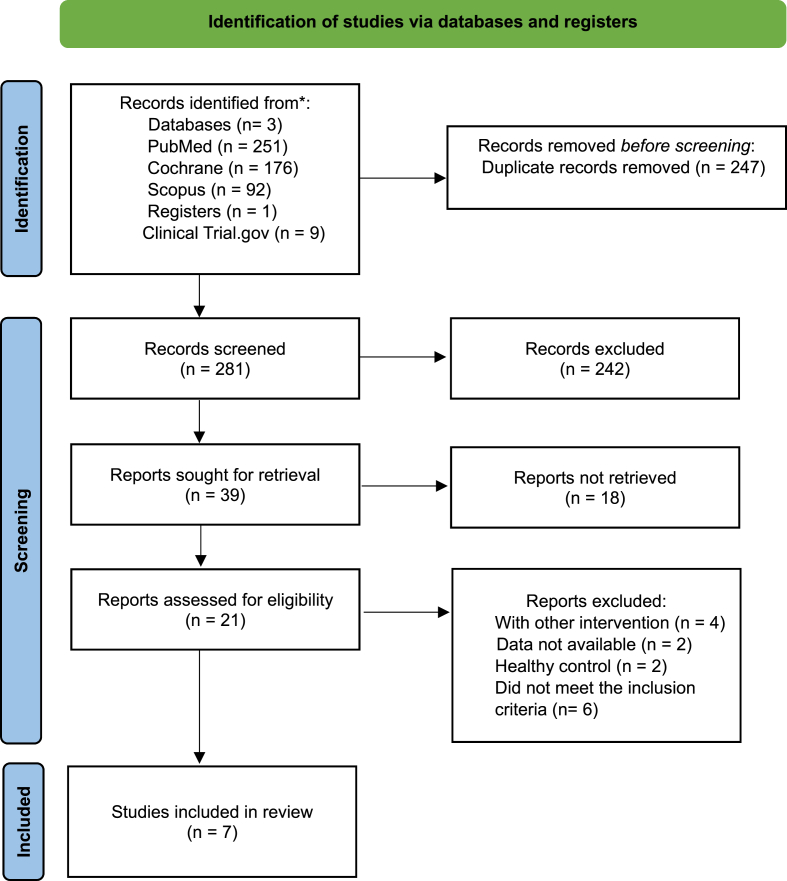
PRISMA Flow chart.

The PRISMA flowchart summarizes the study selection process. A total of 528 records were identified through database searches (PubMed, Cochrane, Scopus, ClinicalTrials.gov). Several yoga-based RCTs were identified during screening but excluded due to non-isolation of pranayama. After removing 247 duplicates, 281 records remained for screening. Based on the title and abstract review, 242 records were excluded. Of the 39 full-text reports sought, 18 could not be retrieved. The remaining 21 reports were assessed for eligibility, of which 14 were excluded due to reasons such as irrelevant interventions (*n* = 4), unavailable data (*n* = 2), healthy controls (*n* = 2), and not meeting the inclusion criteria (*n* = 6). Finally, 7 randomized controlled trials were included in the systematic review and meta-analysis (Fig. 1).

Seven randomized controlled trials comprising 683 participants were included in the final quantitative analysis. Five studies were conducted in India ^9^^,^^10^^,^^15^, ^16^, ^17^, one in Egypt,^18^ and one in the United States of America (USA).^19^ Sample sizes ranged from 60 to 170 participants. All studies included both male and female participants aged 18 years.

Intervention types varied across studies: three studies used alternate nostril breathing 16, 17, 18, two studies used Sheetali pranayama,^9^^,^^10^ one study used bee humming breathing,^15^ and one study used yogic breathing exercises.^19^ Intervention durations ranged from 5 min to 1 h per session, with study periods ranging from single sessions to 3 months. The detailed characteristics of the included studies are presented in Table 1. Across the included trials, pranayama interventions varied in type, duration, and supervision. Intervention duration ranged from short-term programs to several weeks, with most studies employing supervised sessions during the initial phase. The frequency and technique of practice varied across trials, and compliance was primarily assessed through supervised attendance or self-reported adherence.

**Table 1:** Descriptive data of the RCTs reviewed.

Author Name & Year	Country	Study design	Age	Study Population	Total Sample size	Type of intervention & Duration	Outcome assessment	Study Findings
P Shetty et al, 2017^9^	India	Randomized Controlled Trial	25–65 years	Hypertensive patients	*n* = 60 *S* = 30 C = 30	S = Two types of pranayama breathing practices (i.e., Sheetali and Sheetkari) C = quiet sitting Duration: 20 mins everyday, for 30 days	SBP (Mercury Sphygmomanometer), HR, RR, NN50, pNN50, LF band, HF band, and LF/HF ratio	Sheetali and Sheetkari pranayamas appear effective for lowering SBP in individuals with HTN.
Thanalakshmi J et al, 2020^10^	India	Randomized controlled trial	18–60 years	Patients with primary hypertension	*n* = 100 *S* = 50 C = 50	S = Sheetali pranayama C = Without pranayama Duration: 30 min everyday for 3 months	SBP, DBP (Digital BP Monitor), MAP, HR, LF band, HF band, LF/HF ratio, RR Intervals, rMSSD, PP, DoP, and RPP	The intervention group showed a significant reduction in blood pressure and short-term HRV parameters indicating parasympathetic dominance.
Ghati N et al, 2021^15^	India	Single-center, open-label, randomized active-control trial	30–70 years	Patients with essential hypertension	*n* = 70 *S* = 35 C = 35	S = Bee-Humming Breathing C = placebo, slow breathing exercise Duration: 5 min for both groups	SBP, DBP (Digital BP Monitor), MAP, HR, SV, arterial stiffness, TPR, LF band, HF band, LF/HF ratio, SDNN, rMSSD, and pNN50	No significant change in systolic, diastolic, or mean blood pressures after BHB exercise compared to the control group. However, HRV analysis showed increased HF power and decreased LF power during the 5-min BHB recovery phase.
Telles S et al, 2013^16^	India	Randomized Controlled Trial	20–59 years	Participants with essential hypertension	*n* = 90 *S* = 30 C = 60	S = Alternate nostril yoga breathing C = Reading a magazine C = Breath awareness Duration: 10 min	SBP and DBP (Mercury Sphygmomanometer)	The results suggest that the immediate effect of ANYB is to reduce the BP.
Kalaivani S et al, 2019^17^	India	Randomized Controlled Trial	30–60 years	Participants with essential hypertension	*n* = 170 *S* = 85 C = 85	S = Alternate nostril breathing exercise C = Underwent routine treatment Duration: Two times a day (10 min duration of exercise each time) for 5 days, along with routine treatment	SBP, DBP (Digital BP Monitor), HR, and RPP,	The study group showed marked reductions in systolic and diastolic BP, heart rate, and rate-pressure product after 5 days of continuous alternate nostril breathing.
AMA Ismail et al, 2023^18^	Egypt	Randomized Controlled Trial	65 years and above	Older adults with systemic hypertension and high-tension form of primary open-angle glaucoma	*n* = 60 *S* = 30 C = 30	S = received daily alternate-nostril breathing exercise C = did not receive the alternate-nostril breathing exercise Duration: Daily 1 h for four weeks	SBP and DBP	All measurements improved only in the ANBE group. A 4-week ANBE program may serve as an adjunctive therapy to enhance psychological, physiological, and quality-of-life outcomes in older adults with SH and HTF-POAG.
Misra S et al, 2019^19^	United States	3- Arm Randomized Controlled Trial	18–60 years	Patients with uncontrolled hypertension	*n* = 133 *S* = 101 C = 32	S (In-class instruction and YouTube group) = yogic breathing exercises C = no breathing exercises Duration: 15-min yogic breathing exercises practiced 5 times a week for 6 weeks	SBP	Statistically significant systolic blood pressure reduction of at least 5 mm Hg in the intervention group.

**Abbreviations:** SBP - systolic blood pressure; DBP - diastolic blood pressure; MAP - mean arterial pressure; HR - heart rate; SV - stroke volume; TPR - total peripheral resistance; LF - low-frequency; HF - high-frequency; HRV - heart rate variability; RPP - rate pressure product; NN50 - The number of pairs of successive NN intervals that differ by more than 50 ms; pNN50 - proportion of NN50 devided by the total number of NN intervals; RR - respiratory rate; SDNN - standard deviation of RR Intervals; rMSSD - the square root of the mean of the sum of the squares of differences between adjacent normal-to-normal (NN) intervals; PP - pulse pressure; DoP - double product. *Secondary outcomes included SBP, DBP, and heart rate variability parameters where reported.*

### 3.2 Risk of bias assessment

Most studies showed low risk of bias arising from the randomization process, handling of missing outcome data, and measurement of outcomes. Concerns were identified regarding deviations from intended interventions in most studies, with Ghati N et al.,^15^ Telles et al.,^16^ and Kalaivani et al.^17^ demonstrating high risk in this domain. Kalaivani et al.^17^ also showed a high risk for selection of reported results. Overall, one study was rated as low risk (Misra et al.^19^), three studies as having some concerns (Thanalakshmi et al.,^10^ AMA Ismail et al.,^18^ and P Shetty et al.^9^), and three studies as high risk (Ghati N et al.,^15^ Telles et al.,^16^ and Kalaivani et al.^17^) (Supplementary files 1 and 2).

## 4 Outcomes

### 4.1 Primary outcome

**Fig. 2:**
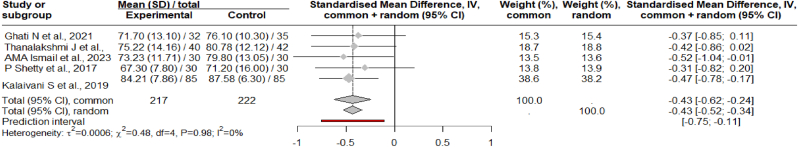
Forest plot comparing heart rate between experimental and control groups across five studies.

The primary outcome of the effect of pranayama on heart rate (HR), was evaluated in five studies (*n* = 439; intervention = 217 and control = 222) that were included in the analysis (Fig. 2). All five studies demonstrated a trend toward lower heart rates in the intervention groups compared to controls. The pooled standardized mean difference was −0.43 (95 % CI: −0.52 to −0.34), indicating a significant reduction in heart rate in the pranayama group. The common-effects model yielded a similar estimate as well (SMD = −0.43, 95 % CI: −0.62 to −0.24). Heterogeneity among the included studies was minimal, with *τ*^2^ = 0.0006, *p* = 0.98, and I^2^ = 0 %. Among individual studies, AMA Ismail et al (2023) showed the largest effect (SMD = −0.52, 95 % CI: −1.04 to −0.01), while P Shetty et al (2017) reported the smallest and least precise effect (SMD = −0.31, 95 % CI: −0.82 to 0.20). The study by Kalaivani S et al (2019) carried the greatest weight (38.2 %) in the analysis due to its larger sample size (*n* = 165) (Fig. 2).

### 4.2 Secondary Outcomes

**Fig. 3:**
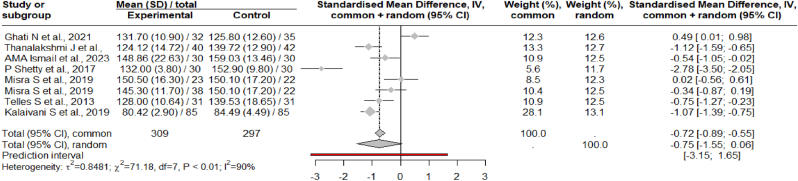
Forest plot comparing systolic blood pressure between experimental and control groups from eight studies.

**Systolic Blood Pressure (SBP):** 7 studies with a total of 606 participants, with 309 individuals randomized to the intervention arm and 297 individuals randomized to the control arm were included (Fig. 3). The overall SMD for systolic blood pressure reduction was −0.75 (95 % CI: −1.55 to 0.06) under the random-effects model. Although the point estimate suggested a moderate reduction in SBP favouring the experimental group, the wide confidence interval indicated that the difference was not statistically significant. In contrast, the common-effects model yielded a significant pooled SMD of −0.72 (95 % CI: −0.89 to −0.55), suggesting a favourable effect of the intervention under the assumption of homogeneity. However, the prediction interval ranged from −3.15 to 1.65, reflecting considerable uncertainty in the true effect across different populations and suggesting potential for both large benefits and no effect in future studies. There was substantial heterogeneity (*τ*^2^ = 0.8481, *p* < 0.01, and I^2^ = 90 %) in the results of the included studies. While P Shetty et al (2017) reported the largest effect in favour of the pranayama intervention (SMD = −2.78; 95 % CI: −3.50 to −2.05), Ghati N et al (2021) observed a small but significant effect favouring the control group (SMD = 0.49, 95 % CI: 0.01 to 0.98), while other studies such as Misra et al (2019) showed borderline results. Overall, a moderate to large, statistically significant reduction in SBP was noted in the common effect model (SMD = −0.72, 95 % CI: −0.89 to −0.5484, *p* < 0.0001) but was marginally non-significant in the random effects model (SMD = −0.75, 95 % CI: −1.5486 to 0.0561, *p* = 0.0638) (Fig. 3).

**Fig. 4:**
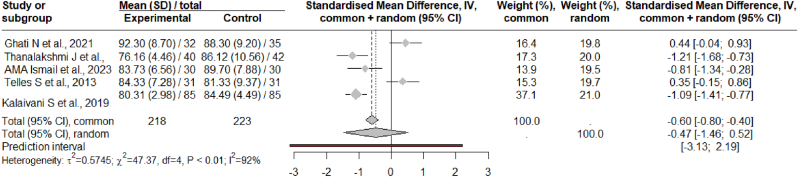
Forest plot showing the effect of interventions on diastolic blood pressure across five studies.

**Diastolic Blood Pressure (DBP):** Among the five studies (*n* = 441; intervention = 218 and control = 223) that were included in the analysis for diastolic blood pressure (Fig. 4), the random-effects pooled SMD was −0.47 (95 % CI: −1.46 to 0.52, *p* = 0.2577), indicating a non-significant reduction in DBP in the intervention group compared to control. In contrast, the common-effects model yielded a statistically significant reduction in DBP with an SMD of −0.60 (95 % CI: −0.80 to −0.40, *p* < 0.0001). At the level of individual studies, Thanalakshmi J et al (SMD = −1.21, 95 % CI: −1.68 to −0.73) and Kalaivani S et al (2019) (SMD = −1.09, 95 % CI: −1.41 to −0.77) reported significant reductions in DBP in favour of the pranayama intervention group, while Ghati N et al (2021) (SMD = 0.44; 95 % CI: −0.04 to 0.93) and Telles et al (2013) (SMD = 0.35, 95 % CI: −0.15 to 0.86) found non-significant increases in DBP. Here too, there was substantial heterogeneity in the studies (I^2^ = 92 %), and a relatively large and uncertain prediction interval (−3.13 to 2.19), indicating that future studies may observe results that span benefit, harm, or no effect (Fig. 4).

In addition to heart rate and blood pressure, some studies also assessed secondary cardiovascular parameters such as heart rate variability indices.

### 4.3 Effect on heart rate variability parameters

To evaluate the effect of pranayama breathing on heart rate, we evaluated the RMSSD between consecutive normal-to-normal (NN) intervals, the proportion of number of pairs of consecutive NN intervals that differ by more than 50 ms (NN50) divided by the total number of NN intervals (PNN50), and high-frequency and low-frequency HRV parameters.

**Fig. 5:**
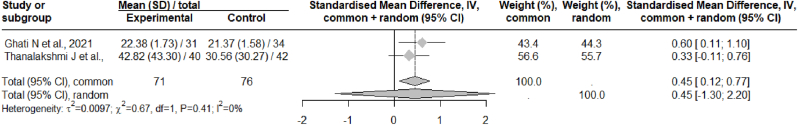
Forest plot comparing rMSSD values between experimental and control groups in two studies.

**The square root of the mean of the sum of the squares of differences between adjacent normal-to-normal (NN) intervals** (**RMSSD):** Data from two studies that were included in this analysis (*n* = 147; intervention = 71, control = 76) indicated a moderate positive effect that was significant only in the common effects model (SMD = 0.45, 95 % CI: 0.1185 to 0.7748, *p* = 0.0076), but not in the random effects model (SMD = 0.45; 95 % CI: −1.30 to 2.20, *p* = 0.41). Heterogeneity was minimal (I^2^ = 0 %) (Fig. 5).

**Fig. 6:**
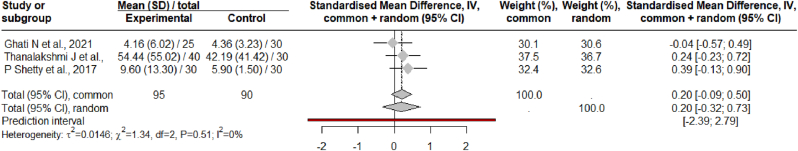
Forest plot comparing PNN50 between experimental and control groups across three studies.

**Proportion derived by dividing NN50 by the total number of NN intervals** (**PNN50):** Analysis of 3 studies (*n* = 185; intervention = 95, control = 90) showed a small, non-significant effect of pranayama under both the random effects model (SMD = 0.2026; 95 % CI [−0.3235; 0.7288]; *p* = 0.2394) and the common effects model (SMD = 0.20; 95 % CI: −0.09 to 0.50; *p* = 0.51). Heterogeneity was absent (I^2^ = 0 %) (Fig. 6).

**Fig. 7:**
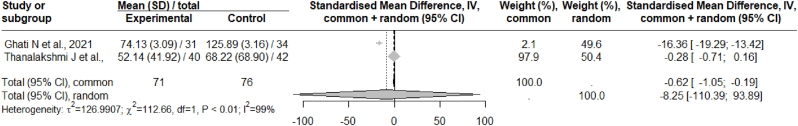
Forest plot evaluating low-frequency power between experimental and control groups in two studies.

**Low Frequency (LF):** Analysis of 2 studies (*n* = 147; intervention = 71, control = 76) showed a significant reduction in LF heartbeats with the common effect model (SMD = −0.6232, 95 % CI: −1.0537 to −0.1927 *p* = 0.0046), but the random effects model revealed no significant effect (SMD = −8.2477, 95 % CI: −110.3868; 93.8915 *p* = 0.4918). However, both studies displayed extremely high heterogeneity (I^2^ = 99.1 %) (Fig. 7).

**Fig. 8:**
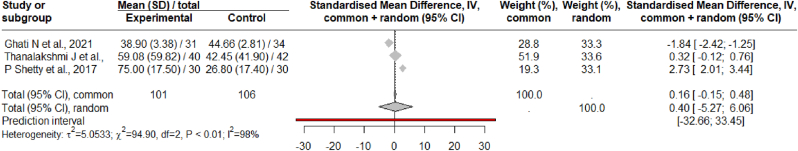
Forest plot comparing high-frequency power between experimental and control groups across three studies.

**High Frequency (HF):** Data from 3 studies (*n* = 207; intervention = 101, control = 106) showed no significant effect of pranayama on HF in either model, with high heterogeneity between studies (I^2^ = 97.9 %) (Fig. 8).

**Fig. 9:**
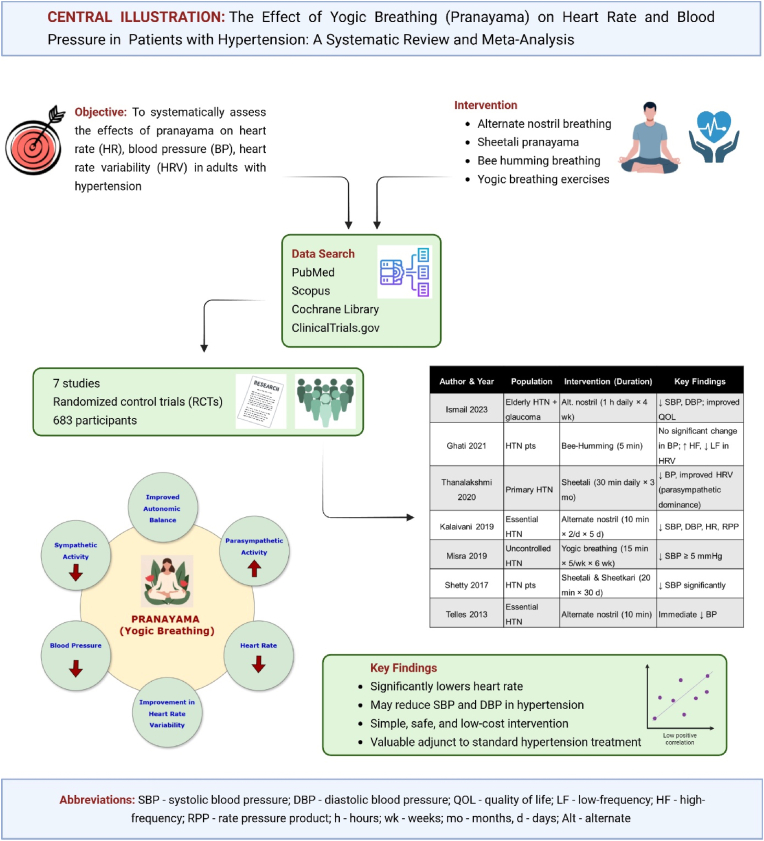
Central illustration summarising the findings of the meta‐analysis.

The results of the meta-analysis are summarised in the central illustration (Fig. 9).

## 5 Discussion

In this comprehensive systematic review and meta-analysis, we demonstrate that pranayama, a form of yogic breathing technique, when practised as a standalone intervention, produces meaningful cardiovascular effects in individuals with hypertension. Our results suggest that pranayama may be an effective non-pharmacological intervention for significantly reducing heart rate and systolic blood pressure. Focusing on pranayama alone allowed clearer attribution of blood pressure and heart rate effects, avoiding heterogeneity from multimodal yoga interventions.

The most consistent finding was a significant reduction in HR across studies, suggesting robust autonomic calming effects of pranayama. From a broad perspective, the physiological mechanisms underpinning these effects are likely multifaceted. It has been demonstrated that pranayama involves slow and controlled breathing, which has been shown to activate the parasympathetic nervous system, leading to reduced sympathetic tone and enhanced vagal activity. This autonomic modulation can result in lowered heart rate and blood pressure. Moreover, pranayama may improve heart rate variability, by allowing for better autonomic regulation.^20^

The relationship between HR and central aortic pressure (CAP) is clinically significant, especially in hypertension management. HR influences arterial wave reflection timing; a slower HR lengthens diastole, improving ventricular filling and delaying wave reflection, thereby reducing CAP.^21^ Although beta-blockers are effective in lowering HR and peripheral blood pressure, they may paradoxically raise CAP. This has been demonstrated in the CAFE (Conduit Artery Function Evaluation) study, which found that atenolol-based regimens were less effective in lowering CAP compared to amlodipine-based therapies, despite similar reductions in brachial pressure.^22^ Conversely, regulation of breathing through pranayama reduces HR through modulation of autonomic tone, by enhancing parasympathetic activity, without eliciting adverse central hemodynamic effects, and can serve as a valuable adjunct therapy for managing hypertension. Our findings align with the physiological rationale for pranayama in that slow and controlled breathing enhances baroreceptor sensitivity, reduces chemoreflex hyperactivity, and improves vascular compliance, all of which are pathophysiologically relevant pathways in hypertension.^23^ The autonomic modulation observed in HR indices supports existing literature describing pranayama's influence on sympathovagal balance.^20^

For blood pressure outcomes, we observed a moderate to large reduction in SBP across studies, though high heterogeneity (I^2^ = 90.2 %) was present. This heterogeneity may be attributable to differences in intervention types, durations, study populations, and measurement methods. Similarly, DBP showed a moderate reduction in the common effect model but not in the random effects model, with substantial heterogeneity (I^2^ = 91.6 %). Regarding HRV parameters, the results were mixed. RMSSD showed improvement in the common effect model only, while PNN50, LF, and HF findings were inconsistent with high heterogeneity in some cases. These variations might reflect methodological differences, population characteristics, or inconsistent intervention protocols across studies. However, the variability in effect sizes across studies evaluating both blood pressure and heart rate warrants cautious interpretation. While some studies reported substantial reductions in blood pressure, others observed minimal or no significant changes. This may stem from differences in study design, participant characteristics, and specific pranayama techniques employed. For example, Sheetali pranayama, characterised by cooling breath techniques, has been associated with significant reductions in blood pressure and heart rate. Conversely, other forms of pranayama may yield less pronounced effects.

In addition to expanding the literature by identifying effects unique to pranayama, our results are consistent with previous systematic reviews assessing breathing-based and yoga-related interventions. In previous meta-analyses, the yoga-based interventions have been shown to significantly lower blood pressure. However, the majority of these studies combined postures, meditation, relaxation, and breathing techniques, making it challenging to pinpoint the active therapeutic component. In such studies, the cumulative effects of several practices are probably responsible for the observed BP reductions rather than breathing alone.

Compared to Garg et al.'s 2024 systematic review and meta-analysis of various breathing techniques, including pranayama, our study focused exclusively on pranayama practices in hypertensive patients. While Garg et al. reported significant reductions in SBP (−12.24 mmHg), DBP (−4.93 mmHg), and HR (−3.16 bpm) with substantial heterogeneity (I^2^ > 78 %),^12^ our meta-analysis demonstrated similar favourable effects on these parameters, though statistical significance varied across models due to high heterogeneity.

Uniquely, our study also included HRV outcomes, offering additional insight into autonomic regulation. In conclusion, while pranayama shows promise as an adjunctive intervention for hypertension, further high-quality research is essential to establish standardized protocols and identify the most effective techniques. Given its low cost, accessibility, minimal side effects, and cultural acceptability, pranayama could serve as a valuable component of a broader hypertension management strategy, particularly in populations hesitant to initiate pharmacological treatments.

### 5.1 Strengths and limitations

Pranayama is an easy-to-perform, low-cost intervention that does not require specialized equipment or infrastructure, making it highly accessible across diverse populations, including those in low-resource settings. Furthermore, the review adheres to PRISMA guidelines, PROSPERO registration, and inclusion of only controlled trials with measurable BP and HRV outcomes. However, this review has several limitations, including heterogeneity in intervention protocols, small sample sizes in many studies, short intervention durations, and potential publication bias. Additionally, the quality of included studies varied, with some exhibiting a high risk of bias. Together, these limitations highlight the need for rigorous, well-designed randomized controlled trials to more conclusively determine the efficacy of pranayama in hypertension management. The use of both mercury and digital BP monitors in the included studies could potentially contribute to measurement variability. Additionally, the exclusion of non-English studies may have restricted the scope of evidence. A formal quantitative subgroup analysis and regression analyses were not feasible due to substantial heterogeneity and inconsistent reporting of intervention characteristics. The strict inclusion of pranayama-only trials may have led to the exclusion of several yoga-based studies and reduced the overall sample size, which may limit the generalizability of the findings.

## 6 Conclusions

This systematic review and meta-analysis suggest that pranayama may serve as a promising adjunctive therapy for reducing heart rate and potentially blood pressure in hypertensive patients. While effects on some parameters remain inconsistent, the overall trend supports the cardiovascular benefits of pranayama. Further high-quality, well-designed, adequately powered large-scale RCTs evaluating pranayama as a standalone intervention with standardised protocols and long-term follow-up are warranted to confirm these findings and better define pranayama's clinical role in hypertension management.

## Funding

Yogapriya Chidambaram, Madhesh Kasi, and Ambalam M. Chandrasekaran were supported by the Yoga-CaRe HF trial funded by the Indian Council of Medical Research (ICMR). Grant number - 50/1/TF-CVD/GIA/2021-NCD-I.

## Declaration of competing interest

The authors declare that they have no known competing financial interests or personal relationships that could have appeared to influence the work reported in this paper.
