# Supplementary figures and images for: The effect of yogic breathing (Pranayama) on heart rate and blood pressure in patients with hypertension: A systematic review and meta-analysis
Source: Indian Heart J. 2026 Jan 22;78(3):135–43. doi: 10.1016/j.ihj.2026.01.004 (PMC13316001; doi:10.1016/j.ihj.2026.01.004)

**Supplementary File 1:** Risk of bias summary


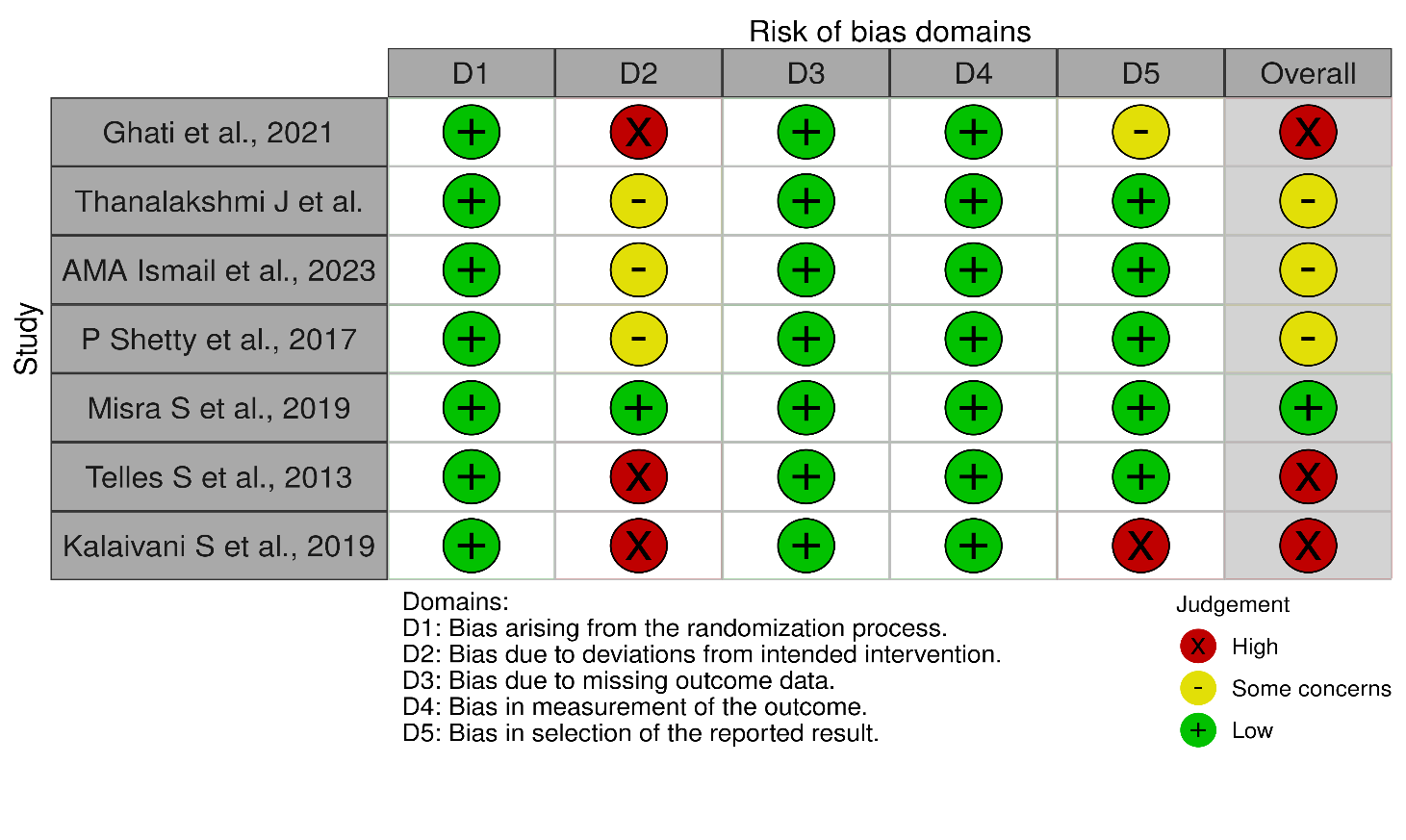


**Supplementary File 2:** Risk of bias graph


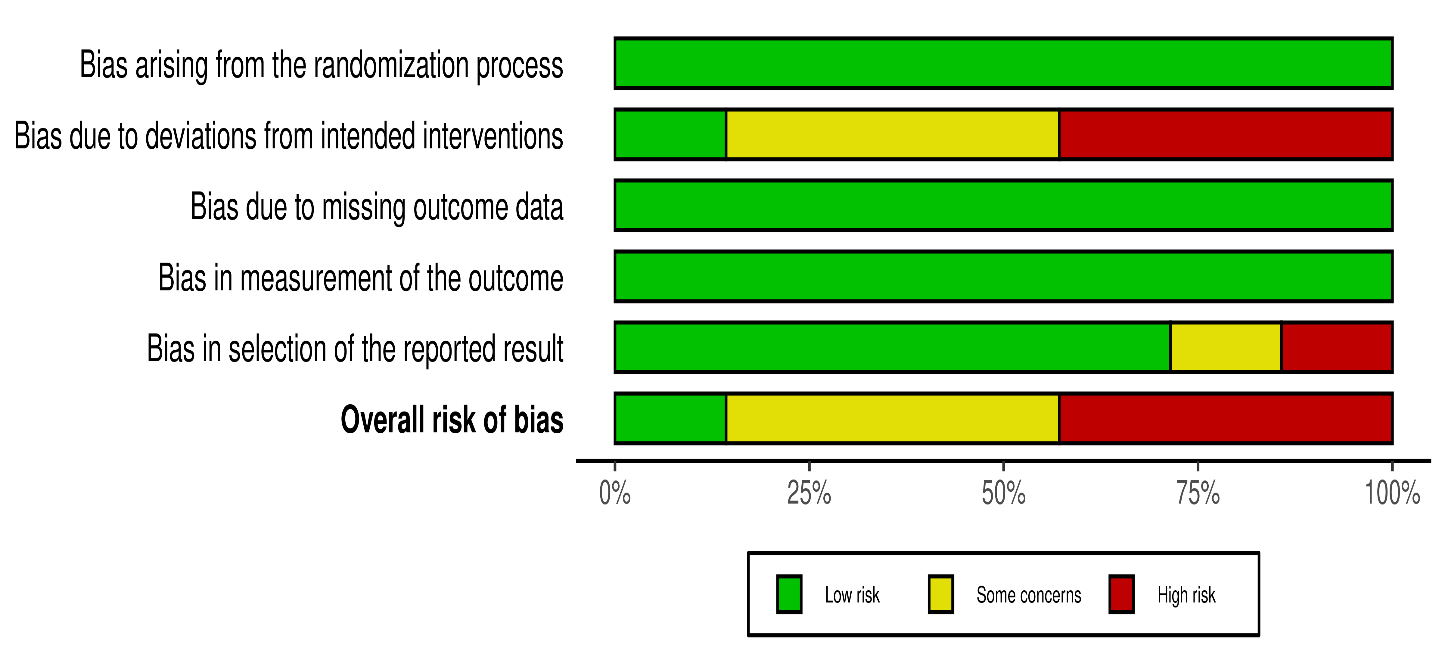

Supplement: Multimedia component 1 [file mmc1.docx]
